# Baicalin Alleviates Chronic Restraint Stress-Induced Depression-like Behavior by Suppressing ROS/H_2_O_2_ Generation via a BDNF-Associated Mechanism in Mice

**DOI:** 10.3390/antiox15010139

**Published:** 2026-01-21

**Authors:** Yu-Ning Teng, Tien-Wei Hsu, Wei-Hao Peng, Cheng-Chun Wu, Tian-Huei Chu, Yung-Kuo Lee, Ming Tatt Lee, Yu-Cheng Ho

**Affiliations:** 1 School of Pharmacy, China Medical University, Taichung City 406040, Taiwan; ynteng@mail.cmu.edu.tw; 2 School of Medicine, College of Medicine, I-Shou University, Kaohsiung 824005, Taiwan; 3 Department of Psychiatry, E-DA Dachang Hospital, I-Shou University, Kaohsiung 807066, Taiwan; 4 Department of Psychiatry, E-DA Hospital, I-Shou University, Kaohsiung 824005, Taiwan; 5 Graduate Institute of Clinical Medicine, College of Medicine, Kaohsiung Medical University, Kaohsiung 807378, Taiwan; 6 School of Medicine, National Tsing Hua University, Hsinchu 300044, Taiwan; 7 Medical Laboratory, Medical Education and Research Center, Kaohsiung Armed Forces General Hospital, Kaohsiung 802301, Taiwan; 8 Office of Postgraduate Studies, UCSI University, Kuala Lumpur 56000, Malaysia; 9 College of Health Sciences, Chang-Jung Christian University, Tainan 711301, Taiwan

**Keywords:** baicalin, depression, chronic stress, hippocampus, long-term potentiation, ROS

## Abstract

Major depressive disorder (MDD) is a leading cause of global morbidity and mortality. Although pharmacological treatments are widely used, their effects are often limited, and nearly half of patients show resistance to current antidepressants, including those unresponsive to all available therapies. These challenges highlight the need to better understand the neurobiological mechanisms driving MDD and to develop novel therapeutic strategies, especially those involving natural compounds with multitarget actions. Baicalin, a bioactive flavonoid from *Scutellaria baicalensis*, exhibits antioxidant, anti-inflammatory, and neuroprotective properties and has recently gained attention for its potential to improve cognitive deficits and mood disorders. In this study, we investigated baicalin’s antidepressant potential and its underlying mechanisms across multiple experimental levels. We found that oral administration of baicalin produced antidepressant-like effects in both naïve mice and those subjected to chronic restraint stress (CRS). CRS impaired hippocampal long-term potentiation (LTP), whereas baicalin restored these synaptic deficits. Importantly, intra-dorsal hippocampal microinjection of the TrkB receptor antagonist ANA-12 abolished baicalin’s antidepressant effects, indicating the involvement of BDNF–TrkB signaling. Baicalin also reduced reactive oxygen species (ROS)/H_2_O_2_ production in a BDNF-associated manner, demonstrating clear antioxidant activity. Molecular docking further suggested that baicalin binds more effectively to the TrkB receptor than ANA-12, supporting its capacity to activate TrkB-mediated signaling. By integrating in vivo, ex vivo, in vitro, and in silico approaches, our study shows that baicalin exerts robust antioxidant in vitro and antidepressant effects in vivo. These benefits are primarily mediated through activation of BDNF–TrkB signaling, leading to reduced ROS/H_2_O_2_ accumulation and alleviation of CRS-induced depression-like behaviors.

## 1. Introduction

Major depressive disorder (MDD) is one of the most prevalent neuropsychiatric conditions and is a leading cause of morbidity and mortality worldwide [1]. According to 2023 survey data from the Global Health Data Exchange, approximately 280 million people suffer from depression globally. MDD is characterized by a persistently low mood, loss of interest or pleasure in daily activities, and recurrent suicidal ideation. Its onset and progression are strongly influenced by stress-related changes in the brain. Notably, early-life adversities have been shown to predispose individuals to depressive symptoms later in adulthood [2]. The etiology of MDD involves a complex interplay of genetic predispositions, environmental exposures, psychological factors, and alterations in multiple neurotransmitter systems. Alarmingly, it is estimated that nearly 60% of individuals who die by suicide have a diagnosis of MDD [3]. Furthermore, about half of MDD patients are resistant to currently available antidepressants, and approximately 20% exhibit a refractory response to all therapeutic interventions [4]. These challenges highlight the urgent need to better understand the neurobiological mechanisms underlying MDD, both to inform the development of novel treatment strategies and to identify reliable biomarkers for monitoring therapeutic responses.

Common treatments for depression include selective serotonin reuptake inhibitors (SSRIs), tricyclic antidepressants (TCAs), and monoamine oxidase inhibitors. However, their therapeutic effects often require several days to appear, and their efficacy is typically short-lived, rarely exceeding 12 weeks [5]. Beyond current pharmacological options, natural products with multitarget properties and favorable safety profiles present promising alternatives [6]. Baicalin (7-glucuronic acid 5,6-dihydroxyflavone), a major bioactive compound extracted from the traditional Chinese herbal medicine *Scutellaria baicalensis* Georgi (Chinese skullcap), possesses potent antioxidant, anti-inflammatory, hypoglycemic, neuroprotective, and anticancer activities [7,8,9]. Recent studies suggest that baicalin enhances anterograde axoplasmic transport in hippocampal glutamatergic neurons, thereby facilitating synaptic vesicle trafficking, improving synaptic function, and alleviating depression-like behaviors [10]. Moreover, bioinformatics analyses combined with animal experiments have demonstrated that baicalin may exert antidepressant effects by regulating neuroinflammation, apoptosis, and oxidative stress [11]. In recent years, baicalin has gained increasing attention for its potential therapeutic value in cognitive impairment, including depression and anxiety. Nevertheless, the precise mechanisms underlying its biological effects remain incompletely understood. Even with currently available antidepressants, patients often experience only partial symptom relief. Thus, there is an urgent need for more effective therapeutic strategies for major depressive disorder. Natural compounds, including baicalin, show promise in alleviating depressive symptoms, attracting significant interest from both the scientific community and the pharmaceutical industry. Therefore, this study aims to investigate the potential of baicalin and related natural compounds in the treatment of depression and to elucidate their underlying mechanisms.

The hippocampus plays a central role in regulating emotions and affective behaviors and has been strongly implicated in the pathophysiology of depression. Studies have shown that chronic stress induces dendritic atrophy and reduces synaptic plasticity in the hippocampus [12], leading to impairments in cognitive and emotional processes and increasing the risk of psychiatric disorders, particularly depression [13]. Previous reports indicate that chronic stress decreases hippocampal brain-derived neurotrophic factor (BDNF) signaling, thereby contributing to depression-like behaviors [14]. Conversely, chronic stress elevates ROS production in the hippocampus, which also promotes depression-like phenotypes [15]. Notably, one study demonstrated that reducing ROS levels while enhancing BDNF expression in the hippocampus produced antidepressant effects, likely through the modulation of oxidative stress and neurotrophic signaling in a PTSD experimental model [16]. Building on these findings, the present study aims to investigate the potential antidepressant effects of baicalin, with a particular focus on its role in regulating neurotrophic factors and oxidative stress in chronic stress-induced depression-like behaviors.

## 2. Materials and Methods

### 2.1. Animals

Adult male C57BL/6 mice (6–8 weeks old; National Laboratory Animal Center, Taipei, Taiwan) were used in this study. The animals were housed in standard cages under a 12 h light/dark cycle (lights on at 6:00 a.m.) with free access to food and water. All experimental procedures were approved by the Institutional Animal Care and Use Committee of the College of Medicine, I-Shou University and conducted in accordance with the ARRIVE guidelines. Every effort was made to minimize the number of animals used.

### 2.2. Drugs

Baicalin (Catalog No. T2775) was purchased from TargetMol (Shanghai, China). ANA-12 (Catalog No. 4781) was purchased from Tocris (Tocris Bioscience, Bristol, UK). A suspension of baicalin or normal saline was administered intragastrically to mice at a dose of 10 or 40 mg/kg/day for four weeks. Control mice that did not receive baicalin were administered normal saline via the same intragastric procedure to control for the effects associated with oral gavage. For ANA-12 administration, bilateral intra-hippocampal microinjections were performed to locally infuse the drug into the dorsal hippocampus.

### 2.3. Chronic Restraint Stress (CRS)

The chronic restraint stress (CRS) procedure was adapted from our previous reports [17,18]. Mice assigned to the CRS group were placed in adjustable cylindrical plastic restrainers that permitted normal respiration but restricted limb movement. They were immobilized for 6 h daily (10:00–16:00) over a 2-week period, then immediately returned to their home cages. Control mice were left undisturbed in their home cages and did not undergo CRS. During the restraint sessions, both CRS and control animals were deprived of food and water. All procedures were designed to minimize discomfort and avoid physical harm.

### 2.4. Stereotactic Cannulation and Drug Infusion

A bilateral intra-hippocampal microinjection experiment was conducted as previously reported [19]. Mice were anesthetized with isoflurane (5% for induction and 2% for maintenance) and positioned in a stereotaxic frame (Stoelting, Wood Dale, IL, USA) on a heating pad maintained at 40 °C. Twenty-four-gauge stainless steel guide cannulae were stereotaxically implanted bilaterally into the dorsal hippocampus (AP: −2.2 mm; ML: ±1.3 mm; DV: −1.3 mm). After at least seven days of recovery from the cannulation surgery, the mice underwent pharmacological manipulations for behavioral evaluation. Drugs were delivered into the dorsal hippocampus via a 30-gauge injection cannula connected to a microinfusion pump (KDS Legato 130; KD Scientific Inc., Holliston, MA, USA). A volume of 0.2 μL drug solution was infused slowly over 5 min, followed by a 5 min hold to prevent backflow. After behavioral testing, mice were euthanized, and cannula placements were verified with cresyl violet Nissl staining. Only animals with confirmed injections accurately localized in the dorsal hippocampus were included in the final analysis.

### 2.5. Behavioral Assessments

#### 2.5.1. Forced Swimming Test (FST)

Behavioral despair was assessed using the forced swim test (FST) as previously described by Peng et al. [18]. Mice were individually placed in a transparent cylinder (20 cm deep) filled with water maintained at 24 ± 1 °C. Immobility was defined as the mouse floating in an upright position with only minimal movements necessary to keep its head above the water. The duration of immobility was recorded during the final 5 min of the 6 min test by an observer blinded to the experimental groups.

#### 2.5.2. Tail Suspension Test (TST)

Behavioral despair was assessed using the tail suspension test (TST) as described by Peng et al. [18]. Mice were individually suspended by the tail using adhesive tape. Each trial lasted 6 min, and immobility—defined as passive hanging with complete motionlessness—was measured during the final 5 min. All assessments were performed by an observer blinded to the experimental groups.

#### 2.5.3. Female Urine Sniffing Test (FUST)

Hedonic behavior was evaluated using the female urine sniffing test (FUST), which leverages rodents’ innate preference for female pheromonal odors [17]. One hour before testing, mice were habituated to a sterile cotton-tipped applicator placed in their home cage. During the test session, mice were first presented with a cotton tip moistened with sterile water for 3 min, and sniffing duration was recorded. After a 60 min interval without the applicator, mice were exposed to a fresh cotton tip infused with female urine for 3 min. All behavioral assessments were performed by an observer blinded to the experimental groups.

#### 2.5.4. Sucrose Preference Test (SPT)

The sucrose preference test (SPT) is a widely used behavioral assay for evaluating anhedonia, a core symptom of depression, in animal models [20]. For adaptation, mice were first given ad libitum access to two bottles containing 1% sucrose solution for 24 h, followed by 24 h with one bottle of 1% sucrose and one bottle of tap water. On the final adaptation day, bottle positions were switched to prevent side preference. After adaptation, all mice were deprived of food and water for 12 h prior to testing. During the SPT, mice were individually housed and provided free access to two bottles—one containing 1% sucrose solution and the other tap water—for 2 h, with bottle positions switched midway to minimize side bias. Fluid consumption was measured by bottle weight, and sucrose preference (%) was calculated as: Sucrose preference (%) = [sucrose consumption (g)/(sucrose consumption (g) + water consumption (g))] × 100%.

#### 2.5.5. Open Field Test (OFT)

The open field test (OFT) was used to evaluate both anxiety-like behavior and locomotor activity [21]. Mice were placed in the center of a white arena (60 × 60 × 70 cm), and their activity was recorded for 10 min using a video tracking system. Locomotor activity was analyzed with EthoVision XT 14 software (Noldus Information Technology, Wageningen, The Netherlands).

### 2.6. Hippocampal Slices Preparations

Coronal hippocampal slices (300 μm) were prepared from adult male C57BL/6 mice following decapitation under 5% isoflurane anesthesia. The slices were allowed to recover for at least 1 h at room temperature before being transferred to a submersion-type recording chamber. One to two slices per mouse were examined for electrophysiological recordings. The chamber was continuously perfused with oxygenated artificial cerebrospinal fluid (aCSF; 95% O_2_, 5% CO_2_) at a rate of 3–4 mL/min. The composition of the aCSF was (in mM): 117 NaCl, 4.5 KCl, 2.5 CaCl_2_, 1.2 MgCl_2_, 1.2 NaH_2_PO_4_, 25 NaHCO_3_, and 11.4 dextrose, adjusted to pH 7.4.

### 2.7. Hippocampal Extracellular Field Recordings

Extracellular recordings were acquired and analyzed using an Axon setup (Axon Instruments, Foster City, CA, USA). Electrophysiological signals were sampled at 5–10 kHz via a MultiClamp 700B amplifier (Molecular Devices, Sunnyvale, CA, USA) and Digidata 1550B AD converter (Molecular Devices, San Jose, CA, USA), controlled with pClamp software (Version 11, Axon Instruments, Scottsdale, AZ, USA), and subsequently analyzed using Clampfit software (Version 11, Axon Instruments). Evoked postsynaptic potentials in the CA1 stratum radiatum were elicited by electrical stimulation (0.02 ms duration) of Schaffer collateral/commissural afferents at 0.033 Hz using a tungsten concentric bipolar microelectrode (WPI TM53CCINS, Sarasota, FL, USA). Field excitatory postsynaptic potentials (fEPSPs) were recorded with glass pipettes filled with aCSF (3–5 MΩ). Synaptic transmission strength was quantified by calculating the fEPSP slope. Long-term potentiation (LTP) was induced using a high-frequency stimulation protocol consisting of four 0.5 s trains of 100 Hz stimuli, separated by 20 s. fEPSPs were monitored for at least 20 min to ensure baseline stability, with the average slope of 10 min of recordings prior to LTP induction defined as the baseline. LTP magnitude was calculated as the average slope of 30 fEPSPs recorded 50–60 min after high-frequency stimulation, expressed as a percentage of baseline. All recordings were performed by an observer blinded to experimental groups.

### 2.8. Cell Lines and Culture Conditions

The PC-12 rat adrenal pheochromocytoma cell line was obtained from the Bioresource Collection and Research Center (BCRC, Hsinchu, Taiwan). PC-12 cells were cultured in RPMI-1640 medium supplemented with 5% fetal bovine serum and 10% heat-inactivated horse serum. The HT-22 mouse hippocampal neuronal cell line was purchased from Ubigene Biosciences (Guangzhou Science City, Guangzhou, Guangdong Province, China) and cultured in DMEM supplemented with 10% fetal bovine serum. All cells were maintained at 37 °C in a humidified incubator with 5% CO_2_.

### 2.9. ROS/H_2_O_2_ Evaluation

The experiments were performed according to the ROS-Glo™ H_2_O_2_ Assay manual (Promega, Madison, WI, USA, Cat. #G8820). Briefly, cells were seeded into a 96-well white plate at a density of 1 × 10^5^ cells per well and incubated with or without the test compounds (baicalin, ANA-12, and menadione) for the designated time. Subsequently, the H_2_O_2_ Substrate Solution was added and incubated at 37 °C for 5 h. The ROS-Glo™ Detection Solution was then added and incubated at room temperature for 20 min. Luminescence was measured using a SpectraMax iD3 multi-mode microplate reader (Molecular Devices, San Jose, CA, USA).

### 2.10. Molecular Docking

Molecular docking analyses were performed using AMDock Version 1.5.2 [22]. The three-dimensional structure of TrkB (PDB ID: 4ASZ) was obtained from the RCSB Protein Data Bank [23]. Structure data files for baicalin and ANA-12 were downloaded from PubChem and converted to PDB format using Open Babel version 2.4.1. ANA-12 is a selective, small-molecule, noncompetitive antagonist of the TrkB receptor. It exhibits a two-site mode of action, involving both high- and low-affinity binding sites, through which it noncompetitively prevents TrkB activation by BDNF [24]. Docking was carried out in AutoDock4 (version 4.2.6) using simple mode with the search space set to automatic. All docking parameters were kept at their default settings, and the resulting interactions were visualized using PyMOL (https://www.pymol.org/).

### 2.11. Statistical Analyses

The sample sizes in this study were determined using G*Power (version 3.1.9.7) (α = 0.05, power ≥ 80%) [25] and were informed by our previous related work [19,21,26]. Data are expressed as means ± SEM and were analyzed using GraphPad Prism 9 (GraphPad Software Inc., La Jolla, CA, USA). Group differences were assessed using one-way ANOVA with Tukey’s post hoc test, while FUST data were analyzed using two-way ANOVA followed by Sidak’s multiple comparisons test; *p* < 0.05 was considered statistically significant.

## 3. Results

### 3.1. Baicalin Exerts Antidepressant-like Effects

**Figure 1:**
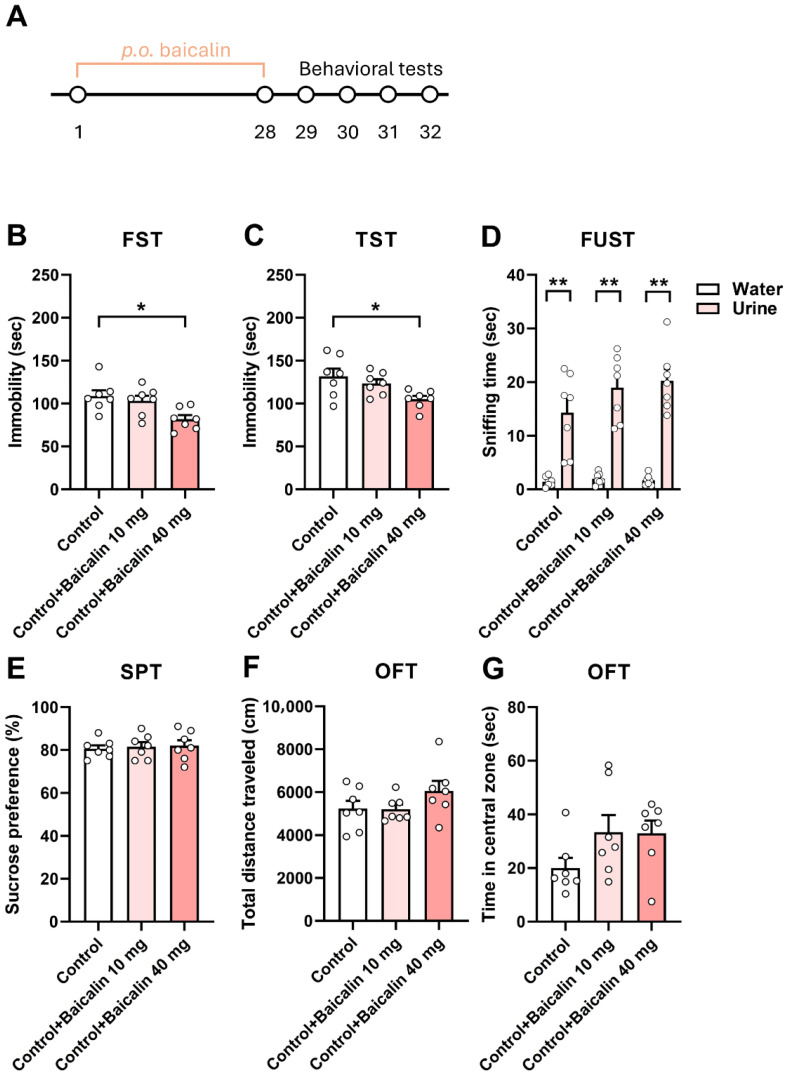
Baicalin produces antidepressant-like effects. (**A**) Experimental design for assessing the behavioral effects of orally administered baicalin in naïve mice. (**B**,**C**) Baicalin (40 mg/kg) significantly reduced immobility time after four weeks of treatment in both the FST (**B**) and TST (**C**). (**D**) Sniffing time in the FUST was unaffected after four weeks of baicalin administration. (**E**) Baicalin did not alter sucrose preference in the SPT following four weeks of oral dosing. (**F**,**G**) Baicalin had no effect on locomotor activity (**F**) or the time spent in the central zone (**G**) in the OFT. (**G**). *n* = 7 per group. * *p* < 0.05 vs. Control in the FST and TST. ** *p* < 0.01 vs. Water in the FUST. FST, forced swim test; TST, tail suspension test; FUST, female urine sniffing test; SPT, sucrose preference test; OFT, open field test. Data are presented as mean ± SEM.

Baicalin has been reported to exert antidepressant effects in several models, including chronic unpredictable mild stress [10,27,28]. However, it remains unclear whether baicalin produces antidepressant effects in chronic restraint stress (CRS)-induced depression-like behaviors, particularly regarding its role in the hippocampus and the underlying mechanisms. Therefore, we first examined whether baicalin produces antidepressant-like effects in naïve mice. We conducted several behavioral assessments: the forced swim test (FST) and the tail suspension test (TST), which measure behavioral despair; and the sucrose preference test (SPT) and female urine sniffing test (FUST), which assess anhedonia. Baicalin was administered orally at doses of 10 and 40 mg/kg for four weeks. Baicalin (40 mg/kg) significantly reduced immobility time in the FST and TST after four weeks of treatment (Figure 1B, one-way ANOVA, F_(2,18)_ = 5.696, *p* = 0.0121; Figure 1C, one-way ANOVA, F_(2,18)_ = 4.650, *p* = 0.0236). It also showed a longer sniffing time for urine compared to water in the FUST (Figure 1D, two-way ANOVA, F_(1,18)_ = 152.2, *p* < 0.01). For the SPT, it maintained a preference for sucrose (Figure 1E, one-way ANOVA, F_(2,18)_ = 0.1169, *p* = 0.8904). By contrast, baicalin had no effect on locomotor activity (Figure 1F, one-way ANOVA, F_(2,18)_ = 1.771, *p* = 0.1985) or time spent in the central zone of the open field test (OFT) (Figure 1G, one-way ANOVA, F_(2,18)_ = 2.211, *p* = 0.1385), indicating that the antidepressant-like effects were not attributable to changes in general activity, but were specific to behavioral despair (FST, TST) and hedonic behavior (SPT, FUST). The effects of baicalin were evident at 40 mg/kg; therefore, this dose was used in subsequent experiments. Collectively, these results suggest that baicalin produces a robust antidepressant-like effect.

### 3.2. Baicalin Alleviates Depression-like Behavior Induced by Chronic Restraint Stress

**Figure 2:**
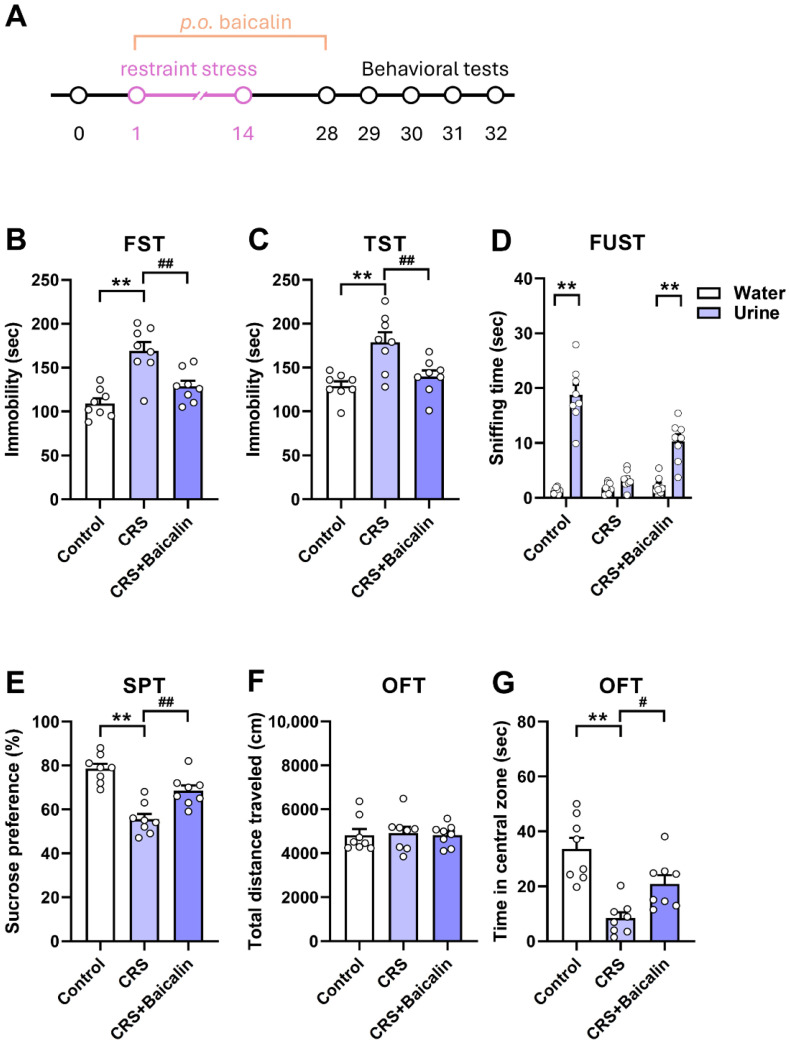
Baicalin exhibits antidepressant-like effects in mice with chronic restraint stress-induced depression-like behaviors. (**A**) Experimental design for evaluating the behavioral effects of orally administered baicalin in mice subjected to daily restraint stress for 14 days. (**B**,**C**) Chronic restraint stress increased immobility time in both the FST (**B**) and TST (**C**), whereas baicalin (40 mg/kg) treatment significantly reduced immobility. (**D**) Chronic restraint stress markedly decreased sniffing time in the FUST, while baicalin treatment significantly increased sniffing time compared with the CRS group. (**E**) Chronic restraint stress significantly reduced sucrose preference in the SPT, whereas baicalin administration restored sucrose preference. (**F**,**G**) Chronic restraint stress did not alter locomotor activity (**F**), but it decreased the time spent in the central zone of the OFT; baicalin administration significantly increased central zone exploration compared with the CRS group (**G**). *n* = 8 per group. ** *p* < 0.01 vs. Control; ^#^ *p* < 0.05, ^##^ *p* < 0.01 vs. CRS in the FST, TST, SPT, and OFT. ** *p* < 0.01 vs. Water in the FUST. CRS, chronic restraint stress. Data are presented as mean ± SEM.

Next, we examined whether baicalin reverses depression-like behaviors induced by CRS, a well-established animal model of depression characterized by behavioral despair and anhedonia [18,21] (Figure 2A). Compared with the control group, CRS-exposed mice showed significantly increased immobility time in the FST (Figure 2B, one-way ANOVA, F_(2,21)_ = 15. 90, *p* < 0.01) and TST (Figure 2C, one-way ANOVA, F_(2,21)_ = 9.797, *p* < 0.01), decreased sniffing time in the FUST (Figure 2D, two-way ANOVA, F_(1,21)_ = 114.4, *p* < 0.01), reduced sucrose preference in the SPT (Figure 2E, one-way ANOVA, F_(2,21)_ = 23.08, *p* < 0.01), and reduced time spent in the central arena of the OFT (Figure 2G, one-way ANOVA, F_(2,21)_ = 14. 94, *p* < 0.01). Oral administration of baicalin (40 mg/kg) for four weeks significantly decreased immobility time in the FST and TST, increased sniffing time in the FUST, enhanced sucrose preference in the SPT, and increased time spent in the central arena in the OFT. Together, these findings indicate that baicalin effectively prevents CRS-induced depression-like behaviors.

### 3.3. Baicalin Restores Chronic Restraint Stress-Induced Impairments in Long-Term Potentiation at Hippocampal Schaffer Collateral–CA1 Synapses

**Figure 3:**
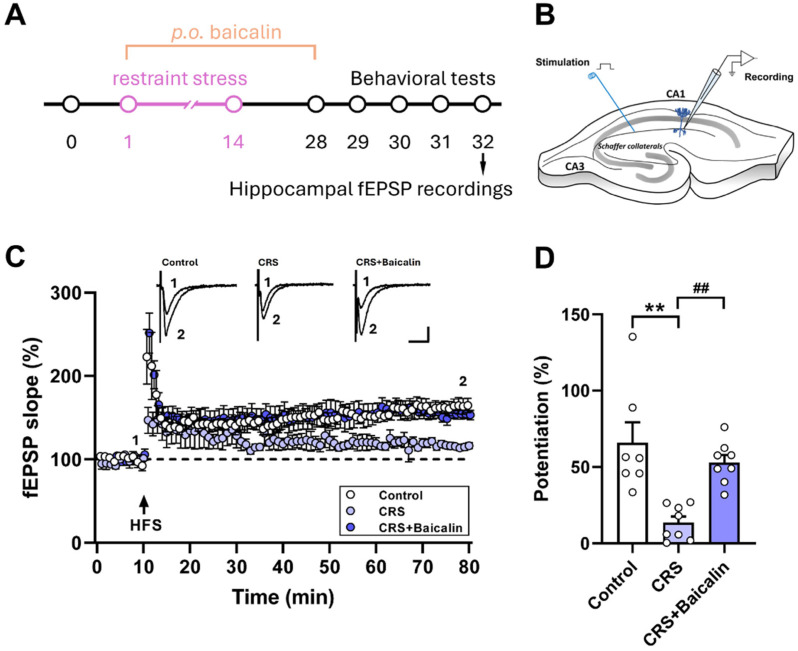
Baicalin reverses chronic restraint stress-induced deficits in long-term potentiation at hippocampal Schaffer collateral synapses. (**A**) Experimental design for assessing synaptic long-term potentiation using extracellular field potential recordings in mice subjected to daily restraint stress for 14 days and treated with oral baicalin. (**B**) Schematic diagram of hippocampal slice field recordings. The stimulation electrode was placed on the Schaffer collateral pathway to deliver electrical pulses, and the recording electrode was positioned in the stratum radiatum of the CA1 region to measure synaptic plasticity in response to Schaffer collateral stimulation. (**C**) Summary of LTP induction in hippocampal CA1 neurons following high-frequency stimulation (HFS; four 0.5 s trains of 100 Hz stimuli) at Schaffer collateral–CA1 synapses. Inset: Representative fEPSP traces recorded at the indicated time points; dashed lines indicate baseline levels. Representative fEPSP traces were taken at the time indicated by number. Calibration: 0.1 mV, 20 ms. (**D**) Summary bar graph showing LTP magnitude measured 70 min after HFS at Schaffer collateral–CA1 synapses. *n* = 7–8 per group. ** *p* < 0.01 vs. Control; ^##^ *p* < 0.01 vs. CRS. Data are presented as mean ± SEM.

Because depression-like behavior has been linked to long-term potentiation (LTP) at Schaffer collateral–CA1 synapses in hippocampal slices [29], we investigated whether chronic restraint stress affects synaptic plasticity at these synapses and whether baicalin provides protection. In control slices, high-frequency stimulation elicited robust LTP (Figure 3C), whereas CRS markedly reduced its magnitude (one-way ANOVA, F_(2,20)_ = 11.90, *p* < 0.01) (Figure 3D). Strikingly, four weeks of baicalin administration (40 mg/kg) preserved LTP, preventing CRS-induced deficits (Figure 3C,D). These findings demonstrate that CRS disrupts synaptic plasticity at Schaffer collateral–CA1 synapses, contributing to cognitive impairment and depression-like behaviors, while baicalin restores plasticity in this hippocampal pathway.

### 3.4. Baicalin Rescues Chronic Stress-Induced Depression-like Behavior Through a Hippocampal BDNF-Associated Mechanism

**Figure 4:**
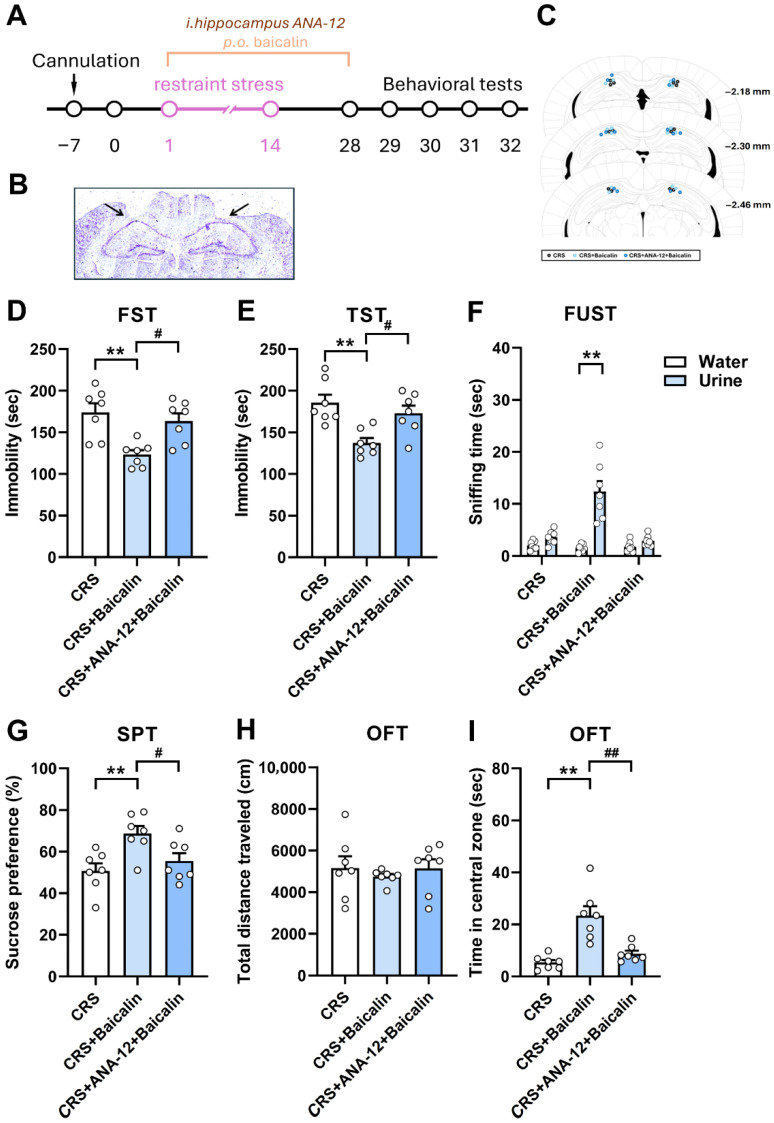
Baicalin reverses chronic restraint stress-induced depression-like behaviors through a BDNF-associated mechanism. (**A**) Experimental design for assessing the behavioral effects of intra-dorsal hippocampal infusion of the TrkB receptor antagonist ANA-12 combined with oral baicalin administration in mice subjected to daily restraint stress for 14 days. (**B**) Representative image showing cannula placement verified by Nissl staining. Arrows indicate the bilateral cannula tip locations. (**C**) Schematic illustration of bilateral cannula tip placements in the dorsal hippocampus for vehicle or ANA-12 microinjection. (**D**,**E**) Baicalin significantly reduced CRS-induced increases in immobility time in both the FST (**D**) and TST (**E**), whereas intra-dorsal hippocampal microinjection of ANA-12 (0.02 nmol) abolished these effects and increased immobility. (**F**) Baicalin significantly increased sniffing time in the FUST compared with the CRS group, whereas ANA-12 microinjection prevented this effect. (**G**) Baicalin significantly restored sucrose preference in the SPT, whereas ANA-12 microinjection blocked this improvement. (**H**) Neither treatment altered locomotor activity (**I**) Baicalin increased central zone exploration in the OFT compared with the CRS group, whereas intra-dorsal hippocampal infusion of ANA-12 prevented this effect. *n* = 7 per group. ** *p* < 0.01 vs. CRS; ^#^ *p* < 0.05, ^##^ *p* < 0.01 vs. CRS + Baicalin in the FST, TST, SPT, and OFT. ** *p* < 0.01 vs. Water in the FUST. Data are presented as mean ± SEM.

Based on previous reports, BDNF is a key protein mediating neuroplastic changes underlying antidepressant effects, particularly for traditional agents such as SSRIs and for newer treatments like ketamine [30,31,32]. To further investigate the mechanism of baicalin’s antidepressant-like effects, we examined whether these actions involve BDNF-associated signaling pathways. To determine whether baicalin exerts its effects within the hippocampus, we specifically targeted this region using microinjection. In this experiment, systemic oral administration of baicalin alleviated depression-like behaviors, as shown by reduced immobility time in the FST (Figure 4D) and TST (Figure 4E), increased sniffing time in the FUST (Figure 4F), enhanced sucrose preference in the SPT (Figure 4G), and increased time spent in the central arena during the OFT (Figure 4I). However, bilateral intra-hippocampal microinjection of the TrkB receptor antagonist ANA-12 (0.02 nmol) abolished the antidepressant-like effects of baicalin, as evidenced by increased immobility in the FST (Figure 4D, one-way ANOVA, F_(2,18)_ = 8.920, *p* < 0.01) and TST (Figure 4E, one-way ANOVA, F_(2,18)_ = 8.712, *p* < 0.01), decreased sniffing time in the FUST (Figure 4F, two-way ANOVA, F_(1,18)_ = 35.68, *p* < 0.01), reduced sucrose preference in the SPT (Figure 4G, one-way ANOVA, F_(2,18)_ = 6.516, *p* < 0.01), and reduced time in the central arena of the OFT (Figure 4I, one-way ANOVA, F_(2,18)_ = 17.30, *p* < 0.01), compared with mice receiving baicalin alone. These findings indicate that baicalin rescues chronic stress-induced depression-like behaviors through hippocampal actions in a BDNF-associated manner.

### 3.5. Baicalin Exerts Antioxidant Effects by Inhibiting ROS/H_2_O_2_ Generation in a BDNF-Associated Manner

**Figure 5:**
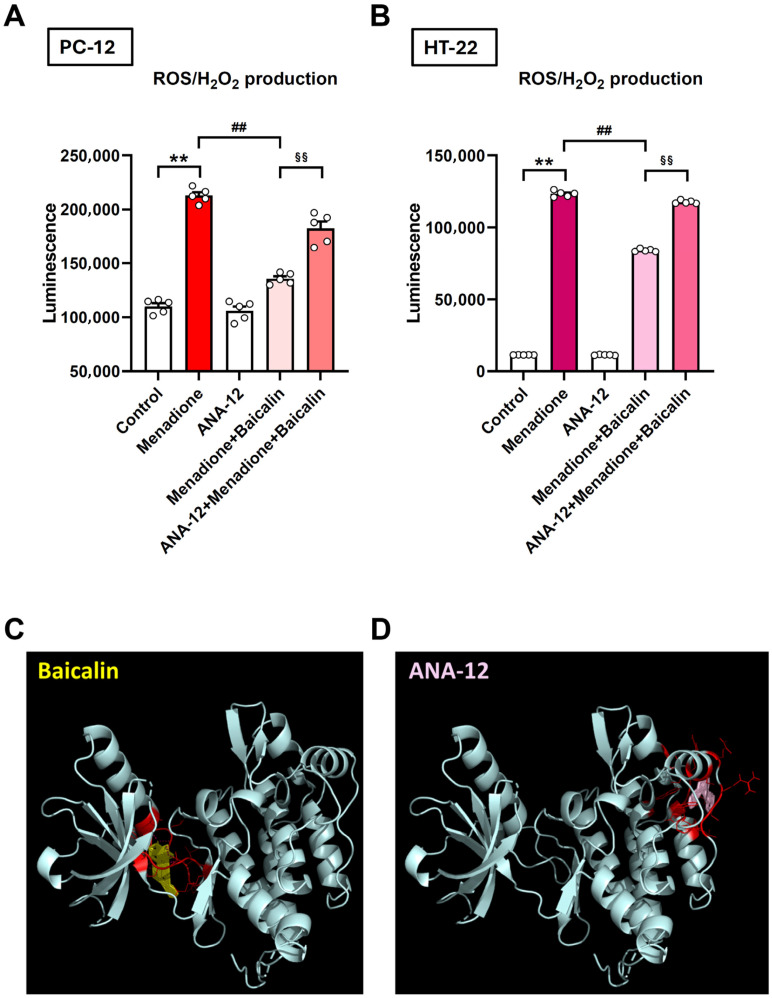
Baicalin demonstrates protective activity against ROS/H_2_O_2_ generation and interacts with a TrkB binding site distinct from that of ANA-12. (**A**) PC-12 cells and (**B**) HT-22 cells were treated with ANA-12 (10 μM) or baicalin (50 μM for PC-12; 10 μM for HT-12; concentration selection based on previous research [33,34]) for 24 h, with or without subsequent exposure to menadione (10 μM, 1 h). Reactive oxygen species (ROS)/H_2_O_2_ generation was measured using luminescence detection. Each experiment was performed at least five times. Notably, baicalin significantly reduced ROS/H_2_O_2_ production, whereas ANA-12 attenuated these antioxidant effects in both neuronal cell lines. (**C**) Molecular docking results showing the interaction between baicalin (yellow) and TrkB, with interacting amino acids highlighted in red. (**D**) Molecular docking results showing the interaction between ANA-12 (pink) and TrkB, with interacting amino acids highlighted in red. ** *p* < 0.01 vs. Control; ^##^ *p* < 0.01 vs. menadione; ^$$^ *p* < 0.01 vs. menadione + baicalin. ROS, reactive oxygen species. Data are presented as mean ± SEM.

Building on the above in vivo and ex vivo findings, we further examined whether baicalin exerts its antidepressant effects by reducing ROS/H_2_O_2_ production and whether these effects depend on BDNF-mediated signaling. To investigate these cellular mechanisms, we employed two cell lines: PC-12 pheochromocytoma cells and HT-22 cells derived from primary mouse hippocampal neurons. In PC-12 cells, baicalin (50 μM) pre-treatment for 24 h significantly attenuated ROS/H_2_O_2_ production induced by the pro-oxidant menadione (10 μM), revealing its antioxidant and protective effects. Notably, ANA-12 (10 μM) abolished the protective actions of baicalin, indicating that BDNF-associated TrkB activation is required for its antioxidant properties (Figure 5A, one-way ANOVA, F_(4,20)_ = 137.4, *p* < 0.01). Similarly, in HT-22 hippocampal neuronal cells, 24 h baicalin (10 μM) pre-treatment significantly reduced menadione-induced ROS/H_2_O_2_ generation (Figure 5B, one-way ANOVA, F_(4,20)_ = 11,974, *p* < 0.01). Consistent with findings in PC-12 cells, ANA-12 effectively blocked baicalin’s protective effects, further implying that its antioxidant action requires BDNF-associated TrkB signaling (Figure 5B). These results indicate that baicalin exerts its antioxidant effects by inhibiting ROS/H_2_O_2_ generation in a BDNF-associated manner.

We next examined the binding interactions of baicalin and ANA-12 with the TrkB receptor using molecular docking analysis. The docking results indicated that baicalin and ANA-12 bind to distinct sites on the TrkB receptor. The lowest binding energies for baicalin and ANA-12 were −12.04 and −6.58 kcal/mol, respectively, with corresponding estimated Ki values of 1.49 nM and 15.03 μM. These findings suggest that baicalin has a markedly higher binding affinity for TrkB than ANA-12. The amino acid residues involved in the predicted interactions were as follows: for baicalin—LEU608, THR609, ASN610, LEU611, GLN612, HIS613, GLU614, ILE616, VAL617, LYS618, PHE619, PHE633, and LYS707; and for ANA-12—PRO738, TRP753, TRP770, CYS781, ILE782, GLN784, ARG786, VAL787, LEU788, LEU803, TRP806, and GLN807 (Figure 5C,D).

## 4. Discussion

Depression is a chronic stress-related disorder characterized by structural and functional alterations in specific brain regions. Clinical studies have shown that patients with MDD exhibit progressive hippocampal atrophy and volume loss [35]. Chronic stress impairs synaptic transmission, a key neuropathological process that contributes to nervous system dysfunction and the development of depression-like behaviors. In this study, we investigated the antidepressant mechanisms of baicalin in a mouse model of depression. Our results demonstrated that baicalin treatment markedly alleviated CRS-induced depression-like behaviors, and this protective effect was associated with its antioxidant activity in the hippocampus. Further experiments revealed that baicalin exerts antioxidant effects and mitigates depression-like behaviors by inhibiting ROS/H_2_O_2_ generation through BDNF-associated signaling. To our knowledge, this is the first evidence that baicalin reduces ROS production and enhances synaptic plasticity in the hippocampus, thereby contributing to its antidepressant effects.

Baicalin, a flavonoid glycoside derived from the roots of *Radix Scutellariae*, is widely recognized as an important component of traditional Chinese medicine [36]. Previous studies have demonstrated that baicalin exhibits several pharmacological properties, including potent antioxidant and anti-inflammatory activities [36,37]. More recently, research has focused on its therapeutic potential in neurological disorders. For example, baicalin has been shown to mitigate hippocampal neuroinflammation in mice by regulating the NF-κB signaling pathway [38]. It also inhibits neuronal apoptosis and activates the BDNF/ERK/CREB signaling cascade, thereby exerting neuroprotective effects that help prevent depression-like behaviors induced by chronic unpredictable mild stress [39,40]. Furthermore, baicalin has been reported to produce antidepressant-like effects in rodent models of depression, such as olfactory bulbectomy-induced depression, by reducing the expression of pro-inflammatory cytokines [41]. In addition, baicalin counteracts the neuroendocrine dysregulation caused by chronic stress, restoring corticosterone homeostasis and alleviating stress-related behavioral disturbances [42]. More recently, baicalin was shown to enhance anterograde axoplasmic transport in hippocampal glutamatergic neurons, thereby improving synaptic function and reducing depression-like behaviors [10]. In the present study, we demonstrated that baicalin produces robust antidepressant-like effects—attenuating behavioral despair and anhedonia—in a mouse model of chronic restraint stress. Moreover, our ex vivo electrophysiological recordings revealed that baicalin restored LTP at Schaffer collateral–CA1 synapses, suggesting a direct improvement in hippocampal synaptic plasticity. These restorative effects may be mediated by baicalin’s ability to reduce ROS production, which in turn enhances BDNF accumulation and promotes neuroprotective signaling. Collectively, our in vitro, ex vivo, and in vivo findings provide converging evidence that baicalin exerts antioxidant in vitro and antidepressant effects in vivo through the preservation of hippocampal function. These findings highlight baicalin as a promising therapeutic candidate for preventing or alleviating stress-related mood disorders.

The hippocampus, particularly the CA1 region, is critically involved in emotional regulation and is highly vulnerable to stress-induced damage [43,44,45]. As a central structure for both memory and emotion, it contains abundant stress hormone receptors, such as the glucocorticoid receptor. Prolonged exposure to stress can disrupt hippocampal structure and function, leading to impaired emotional processing and an increased risk of mood disorders, including depression and anxiety [43,46]. A large body of evidence implicates abnormalities in hippocampal synaptic structure and function in the pathophysiology of depression [10,47]. In the present study, we employed ex vivo field electrophysiological recordings to assess synaptic transmission in the hippocampal CA1 region. We observed that CRS impaired HFS-induced LTP, a well-established cellular correlate of learning, memory, and synaptic plasticity, whereas baicalin treatment significantly reversed this impairment. These findings provide mechanistic support that baicalin may exert its antidepressant effects through restoration of hippocampal synaptic plasticity. More broadly, our results reinforce the concept that enhancing hippocampal function—by preserving neuronal activity and promoting synaptic plasticity—can alleviate emotion-related behavioral abnormalities, including depression-like behaviors [48].

**Figure 6:**
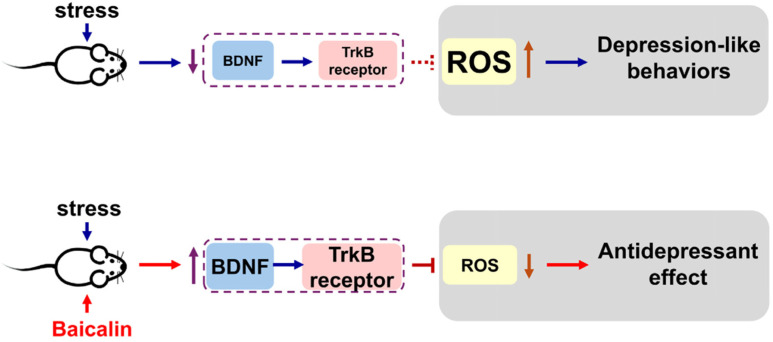
A schematic model illustrating the cellular mechanisms by which baicalin mitigates CRS-induced depression-like behavior in the hippocampus. The diagram depicts the proposed mechanism through which baicalin protects against hippocampal ROS/H_2_O_2_ generation. Chronic restraint stress induces depression-like behaviors in mice (**upper panel**), whereas baicalin alleviates these behaviors (**lower panel**). This antidepressant-like effect is suggested to result from reduced ROS/H_2_O_2_ production and enhanced antioxidant activity. The antioxidant effect of baicalin is mediated through activation of the TrkB receptor. Consequently, baicalin activates the BDNF–TrkB signaling pathway, decreases ROS/H_2_O_2_ accumulation, and ultimately produces antidepressant effects.

Chronic stress exposure induces multiple pathological alterations in the brain, including elevated oxidative stress, disrupted synaptic transmission, and neurochemical imbalances [42,49]. Oxidative stress arises from an imbalance between the generation of ROS and the antioxidant defense systems of neural cells. Among brain regions, the hippocampus is particularly vulnerable to chronic stress-induced oxidative damage [50,51]. Previous studies have shown that elevated corticosterone levels promote ROS production, contributing to depression-like behaviors in chronic unpredictable mild stress (CUMS) models [52]. Conversely, inhibition of ROS accumulation in the hippocampus alleviates CUMS-induced depressive behaviors, underscoring the critical role of hippocampal antioxidant capacity in emotional resilience [53]. Excessive ROS accumulation not only impairs neuronal integrity but also suppresses BDNF expression, creating a vicious cycle in which reduced BDNF exacerbates oxidative stress and promotes depressive pathology [54,55]. BDNF, a key neurotrophin regulating synaptic plasticity, has been extensively implicated in the pathophysiology of depression; its dysregulation leads to impaired synaptic connectivity and reduced excitatory neurotransmission [56]. Elevated hippocampal BDNF levels have been observed in patients receiving antidepressant treatment compared with untreated individuals [57]. Moreover, pharmacological enhancement of antioxidant activity, such as through carvedilol administration, increases brain BDNF levels and produces antidepressant-like effects in stress-exposed mice. Similarly, activation of BDNF-mediated pathways reverses lipopolysaccharide-induced depressive behaviors by upregulating antioxidant enzymes (heme oxygenase-1 and NAD(P)H quinone dehydrogenase 1) and suppressing pro-inflammatory gene expression [58]. In our in vitro experiments, menadione exposure elevated ROS/H_2_O_2_ production in both PC12 and HT-22 cells, whereas baicalin treatment significantly attenuated this effect. The protective action of baicalin was abolished by the TrkB receptor antagonist ANA-12, indicating that its neuroprotective effects are mediated through a BDNF–TrkB-dependent mechanism. Consistently, our in vivo findings showed that the antidepressant-like effects of baicalin were eliminated by intra-hippocampal microinjection of ANA-12. Moreover, our molecular docking analysis revealed that baicalin and ANA-12 bind to distinct sites on the TrkB receptor, and that baicalin exhibits a substantially higher binding affinity than ANA-12. These findings suggest that baicalin interacts with the TrkB receptor more effectively. Although these in silico docking results suggest the proposed binding energies and Ki values, they are computational estimates. The different binding sites for baicalin and ANA-12 revealed the partially reversing effects of ANA-12 on the protective phenomenon of baicalin in terms of ROS production. These results suggest that baicalin interacts more effectively with the TrkB receptor to exert its functions, including both antioxidant and antidepressant effects. Nevertheless, the present study only detects the H_2_O_2_ production, which represents only one component of oxidative stress. And the long-term in vitro effects were not included. In addition, the use of PC-12 and HT-22 does not fully represent primary hippocampal neurons. These limitations warrant future examination. Together, these findings demonstrate that baicalin restores redox homeostasis and synaptic plasticity through activation of the BDNF–TrkB signaling pathway, highlighting its potential as a promising therapeutic agent targeting oxidative stress-related synaptic dysfunction in depression (Figure 6).

## 5. Conclusions

We integrated behavioral assessments in vivo, electrophysiological recordings ex vivo, ROS/H_2_O_2_ analyses in vitro, and molecular docking in silico to elucidate the mechanisms underlying baicalin’s antidepressant and antioxidant actions. Our findings demonstrate that baicalin exhibits potent neuroprotective effects, driven primarily by the activation of BDNF–TrkB signaling. This activation reduces ROS/H_2_O_2_ accumulation and ameliorates CRS-induced depression-like behaviors. Together, these results highlight baicalin as a promising neuroprotective candidate for developing novel antidepressant strategies targeting oxidative stress-related neuropsychiatric disorders.

## Data Availability

The original contributions presented in this study are included in the article. Further inquiries can be directed to the corresponding authors.
